# Effects of Internal Fixation for Mid-Shaft Clavicle Fractures on Shoulder Kinematics During Humeral Elevations

**DOI:** 10.3389/fbioe.2021.710787

**Published:** 2021-07-22

**Authors:** Li-Wei Hung, Hsuan-Yu Lu, Chung-Hsun Chang, Tsan-Yang Chen, Ting-Ming Wang, Tung-Wu Lu

**Affiliations:** ^1^Department of Biomedical Engineering, National Taiwan University, Taipei, Taiwan; ^2^Department of Orthopedic Surgery, Shin Kong Wu Ho-Su Memorial Hospital, Taipei, Taiwan; ^3^Department of Orthopedic Surgery, National Taiwan University Hospital, Taipei, Taiwan; ^4^Department of Orthopedic Surgery, School of Medicine, National Taiwan University, Taipei, Taiwan

**Keywords:** motion analysis, range of motion, scapula, clavicle fracture, locater

## Abstract

**Background:**

Mid-shaft clavicle fractures account for 35 to 44% of injuries to the shoulder girdle. There is increasing evidence to support surgical repair, but poor functional outcomes have been reported, and associated factors remain unclear.

**Methods:**

The three-dimensional poses of the shoulder bones during arm elevations were measured in 15 patients treated for mid-shaft clavicle fractures by open reduction and internal fixation, and in 15 healthy controls.

**Results and Conclusion:**

No significant between-side differences were found in the clavicle length after surgery (*p* > 0.05). The patients showed increased scapular protraction at lower elevation angles and reduced scapular retraction at higher elevation angles during frontal-plane elevations, with significantly reduced clavicle retraction (*p* < 0.05), with unaltered scapular rotation and tilt. The ranges of the observed changes were reduced to arm elevations at 60° and 90° in the scapular and sagittal planes. Similar changes were also found on the unaffected side, suggesting symmetrical bilateral compensation. The results suggest that shoulder kinematics in multi-plane arm elevations should be monitored for any signs of compromised bone motions following surgical treatment, and that rehabilitative training may be needed on both sides to improve the bilateral movement control of the shoulder complex.

## Introduction

Clavicle fractures account for 2.6 to 10% of all adult fractures and 35 to 44% of injuries to the shoulder girdle. The reported annual incidence of clavicle fractures in adults ranges from 0.29 to 0.64 per 1,000 population per year (Khan et al., 2009; Hsiao et al., 2012). The injury is classified into three types following the classification system described by Allman according to the anatomical site of fracture (Allman Jr., 1967) and modified by Robinson according to the fracture comminution and ligamentous injury (Khan et al., 2009).

Mid-shaft clavicle fractures have traditionally been managed through conservative treatment such as a simple sling or figure-eight bandages (Neer II, 1960; Rowe, 1968). Being an unstable fracture, the gap of a mid-shaft clavicle fracture often increases progressively while the sternocleidomastoid muscle pulls the medial segment of the fracture superiorly, and the arm’s weight and the pectoralis major muscle pulls the lateral segment inferiorly and medially (Mark and Lazarus, 2006). Therefore, the best result with conservative treatment would be fracture unification at the initial displaced position, often with inferior and medial displacement of the lateral fragment (McKee et al., 2003). The resulting clavicle shortening deformity is associated with scapular dyskinesis, which may be related to the reported increased risk of persistent shoulder disability (Eskola et al., 1986; Mark and Lazarus, 2006; Postacchini et al., 2010) and unsatisfactory clinical results (Hill et al., 1997). Shortening deformity of the clavicle was found to place the scapula in a more anteriorly tilted and internally rotated (protraction) position (Ledger et al., 2005; Kibler and Sciascia, 2010). An anteriorly tilted scapula may further result in subacromial impingement, leading to a subsequent rotator cuff tear leading to cuff arthropathy (Ludewig and Cook, 2000). Altered alignment and movements of the scapula in scapular dyskinesis are accompanied by modified lines of action and tension of the muscles affecting their efficiency (Myers et al., 2005). Overall, such deviations of skeletal alignment owing to malunions of the clavicle have been found to reduce shoulder function as revealed by self-administered questionnaires (Eskola et al., 1986; Hill et al., 1997; Ledger et al., 2005; Mirzatolooei, 2011). Previous studies evaluating the effects of clavicle malunion or shortening on the skeletal alignment of the shoulder complex used mainly medical imaging methods at standard postures (Hill et al., 1997; McKee et al., 2003; Ledger et al., 2005; Lazarides and Zafiropoulos, 2006; Mirzatolooei, 2011). Owing to technical difficulties, more detailed information about the adaptations of the shoulder complex that individuals with malunions of the clavicle usually use to perform tasks is limited (Kontaxis et al., 2009).

There is increasing evidence to support surgical repair to maintain the anatomical structure of the clavicle considering the limitations of conservative treatment. A recent study suggests that a subset of mid-shaft clavicle fractures may be best treated with primary surgical treatment to reduce the incidence of non-union and symptomatic malunion while providing improved functional results (Bernstein, 2007). Statistically significant improvements in return-to-activities and time-to-union have also been associated with primary surgical management of displaced middle-third clavicle fractures (Morgan et al., 2010). Among the existing surgical treatments for mid-shaft clavicle fractures, plate fixation is currently the standard (Ferran et al., 2010), primarily owing to the rigid fixation design and the ability to restore rotation deformity. However, despite the application of stable implants such as a locking plate, poor functional outcomes of the shoulder complex primarily related to scapular dyskinesis after surgical treatment for clavicle fractures have also been reported (Mirzatolooei, 2011; Robinson et al., 2013; Shields et al., 2015). Scapular dyskinesis is characterized by altered position and motion of the scapula with modified lines of action and tension of the muscles or reduced muscle strength or even impaired motor control, affecting the performance of functional activities of daily living such as brushing the hair on the same side, answering the telephone and eating soup with a spoon (Nelson, 1997; Tang et al., 2001). Patients with scapular dyskinesis have worse functional outcomes compared with those without scapular dyskinesis (Shields et al., 2015). While scapular dyskinesis is associated with poor functional outcomes, the specific changes in the alignment and movements of the scapula during shoulder movement in patients with surgically treated mid-clavicle fractures have not been well documented. A detailed quantitative study on the kinematics of the individual bones of the shoulder complex will help reveal these underlying changes after surgical treatment.

Measurement of the kinematics of the bones of the shoulder complex is technically challenging, primarily because of the extensive movement of the scapula underneath the skin as the shoulder performs large movements. The small and tubular shape of the clavicle is also a major factor contributing to the difficulty of kinematic measurement. Previous studies have used magnetic tracking sensors (Lukasiewicz et al., 1999; Borstad and Ludewig, 2002; McClure et al., 2004; Rundquist and Ludewig, 2004) and inertial measurement units (IMU) (Cutti et al., 2008, 2014; Parel et al., 2012) to measure the three-dimensional (3D) kinematics of the scapula during various upper limb movements. These sensors were placed either on the acromion (Borstad and Ludewig, 2002; Rundquist and Ludewig, 2004) or the spine of the scapula (McClure et al., 2004; Cutti et al., 2008, 2014; Parel et al., 2012), but both approaches are subject to skin movement artifacts (Forner-Cordero et al., 2008; Weenk et al., 2013; Teufl et al., 2019). Even though the measurement accuracy of the magnetic sensors has been assessed by bone pin data (Karduna et al., 2001), it is noted that the skin movement may be limited by the bone pins (Ramsey et al., 2003; Cappozzo et al., 2005). Electromagnetic tracking devices are also susceptible to nearby metal interference (Lugade et al., 2015). IMUs have been assessed for accuracy by using an optoelectronic system during functional tasks, giving RMS errors of less than 3.6° (Cutti et al., 2008), which may be attributed to the so-called inertial sensor drift (Luinge et al., 2007) and linear acceleration interference (Zhang et al., 2012) apart from skin movement artifacts. On the other hand, while these sensors provide the pose (i.e., position and orientation) of the underlying scapula, determining the relationship between the sensor coordinate system and the scapular anatomical coordinate system is not straightforward. This often requires identifying the positions of the bony anatomical landmarks via a digitization procedure or using an experimental procedure to ensure the alignment between the coordinate systems. For magnetic tracking devices, Poppen and Walker (1976) used a single point locater attached to a 3D spatial linkage instrument to identify sequentially the spatial positions of three bony landmarks on the scapula, i.e., the acromial angle (AA), the root of the scapular spine (RS) and the inferior angle (IA). However, the need to identify the positions of the three bony landmarks at each scapular position made the method too time-consuming for clinical use (Johnson et al., 1993). A three-pointed scapular locator which can be applied over the three bony landmarks was developed to measure the scapular kinematics during static positions using a magnetic tracking device (Johnson et al., 1993; Meskers et al., 1998; Karduna et al., 2001). The alignment of sensor and scapular coordinate systems via experimental approaches is also quite challenging because their relative positions are changing during the movement owing to the soft tissue movement (Cutti et al., 2005, 2006; de Vries et al., 2010). Multiple calibrations over the movement ranges may be needed to reduce the sensor/bone misalignment during shoulder motions (Zabat et al., 2019). On the other hand, few studies include the clavicle owing to the physical dimensions of the clavicle, which prevented the sensors from being securely and comfortably attached over the clavicle (Cutti et al., 2008). Difficulties also include the encumbrance of multiple sensors or wires when measuring the motions of the shoulder bones.

Skin marker-based stereophotogrammetry is free from interferences by nearby metal objects or difficulties with small bones, multiple sensors, or wires as in skin-mounted sensors. It has been widely available for 3D measurement of human movement. However, its applications in the measurement of shoulder and upper extremity motions have been less than those in the lower extremities, primarily owing to the difficulties related to the extensive movement of the scapula underneath the skin (Cutti et al., 2005). For the kinematics of the upper extremities, the motions of the scapula and clavicle have been ignored or simplified, and the motions of the humerus relative to the trunk have been traditionally taken as the motions of the shoulder complex in most skin marker-based measurements (Cutti et al., 2005; Garofalo et al., 2009). Several approaches have been proposed to improve this condition, including standardized marker set and calculation methods recommended by the International Shoulder Group (Wu et al., 2005; Kontaxis et al., 2009), and similar methods using regression equations to obtain 3D scapular poses (de Vries et al., 2010). The use of a semi-rigid marker cluster placed directly on the flat surface of the posterior part of the acromion was also proposed to measure the motions of the scapula during arm movement (Merolla et al., 2019). However, the cluster is still subject to movements relative to the underlying scapula. For fast and accurate measurement of the 3D scapular poses for clinical applications, a three-pointed scapular locator applied over the three bony landmarks was developed to measure the scapular kinematics during static positions with optical motion capture systems (Kuo et al., 2018). This locator has been shown to have high test-retest reliability (Kuo et al., 2018) and is a useful tool for measuring the pose changes of the bones of the shoulder complex in patients with mid-shaft clavicle fractures after surgery, which has not been documented in the literature.

The purpose of the current study was to quantify the effects of internal fixation for mid-shaft clavicle fractures on the 3D bone poses of the shoulder complex during multi-plane elevations using computerized optical motion analysis with the new three-pointed scapular locator. It was surmised that the underlying mechanisms responsible for the compromised functional performance in these patients could be revealed at the level of the individual bones of the shoulder complex during standardized elevation movements compared to healthy controls.

## Materials and Methods

**FIGURE 1:**
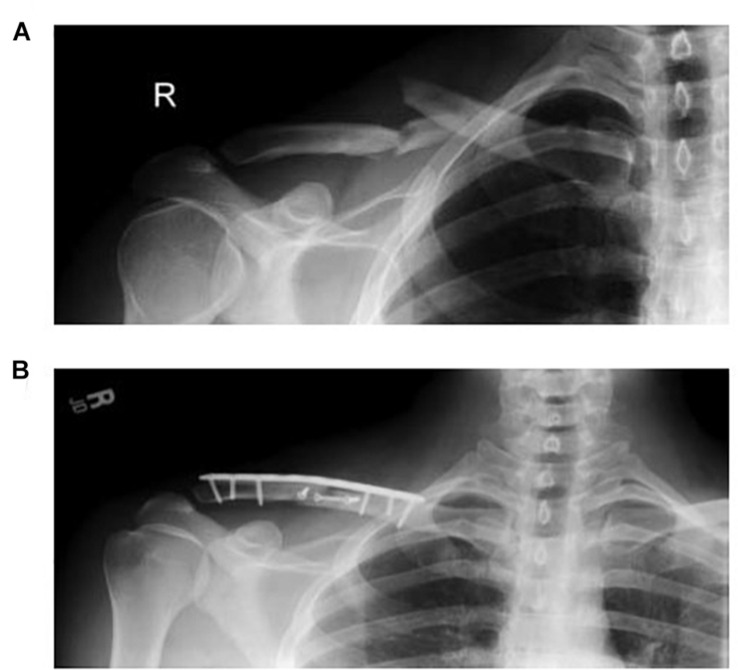
Radiographs of a typical patient treated for a mid-shaft clavicle fracture by open reduction and internal fixation before **(A)** and after **(B)** surgery.

Fifteen patients treated for mid-shaft clavicle fractures by open reduction and internal fixation (Surgical group; 5 females and 10 males; age: 31.4 ± 11 y/o; and BMI: 23.9 ± 4 kg/m^2^) were recruited from the Department of Orthopedic Surgery, National Taiwan University Hospital from July 2014 to June 2016 to participate in the current study. Patients were included if they were aged between 18 and 50 years, had undergone open reduction and internal fixation surgery for a displaced mid-shaft clavicle fracture, and had complete fracture union at 6 months post-surgery (Figure 1). A subject was excluded if he/she had open or pathological fractures, displaced mid-shaft clavicle fractures not healed at 6 months, previous injuries of the upper extremity, multiple trauma, impaired neurological function, or injuries associated with the acromioclavicular or sternoclavicular joint. Following surgery, the patients were referred to the physical therapy department for rehabilitation of the affected side following an 8-week protocol (Pecci and Kreher, 2008). Sling immobilization was maintained for comfort for 1 to 2 weeks and could be discontinued when the major pain subsided. The patients started range-of-motion pendulum exercises as soon as the pain allowed and progressed gradually to active range-of-motion and strengthening exercises over 4 to 8 weeks. The physical therapist determined the duration of immobilization and the dosage of the active exercises according to the patient’s condition. Another 15 healthy controls (Control group; age: 23.3 ± 2 y/o; BMI: 24 ± 2 kg/m^2^) without previous trauma to either shoulder were also recruited to match with the Surgical group in sex, age, and BMI. All the subjects provided informed written consent as approved by the Institutional Review Board.

The plain radiographs of the injured shoulders for all the patients were reviewed by two senior orthopedic surgeons (LWH and TMW), and the diagnosis of mid-shaft clavicle fracture and the evidence of union were made by consensus. All the patients enrolled in the current study had been treated in a standardized way with an infraclavicular incision and a myo-periosteal flap elevated from the fracture segments. Fixation was performed using the Acumed clavicle fixation system (Hillsboro, Oregon, United States), consisting of a pre-contoured titanium locking plate with 2.7 mm or 3.5 mm screws. All surgical procedures were performed under general anesthesia by a single orthopedic surgeon (LWH). The affected arm was placed in an arm sling and standard rehabilitation was arranged first day post-surgery.

Each subject in the Surgical group received a computed tomography (CT) scan of both shoulders after the union of the fracture for the 3D reconstruction of the shoulder complex, from which the union, length, percentage of shortening, and the degree of angulation deformity of the clavicle were measured. Subjective outcome at the time of the experiment was assessed via DASH score (Gummesson et al., 2003), Constant score (Constant and Murley, 1987), VAS score, and the American Shoulder and Elbow Surgeon questionnaire (ASES score; Richards et al., 1994). *A priori* power analyses based on results of a pilot study on 4 patients and 4 healthy subjects using G^∗^POWER software (Erdfelder et al., 1996) determined that 12 subjects for each group would yield a power of 0.8 at a significance level of 0.05 with a large effect size (Cohen’s *d* = 0.9–3.8) for the comparisons of the clavicular and scapular orientations during arm elevation between the Surgical and Control groups using a two-group independent *t*-test, and between sides of the Surgical group using a paired *t*-test. Thus, 15 subjects for each group were considered adequate for the main objectives of the current study.

**FIGURE 2:**
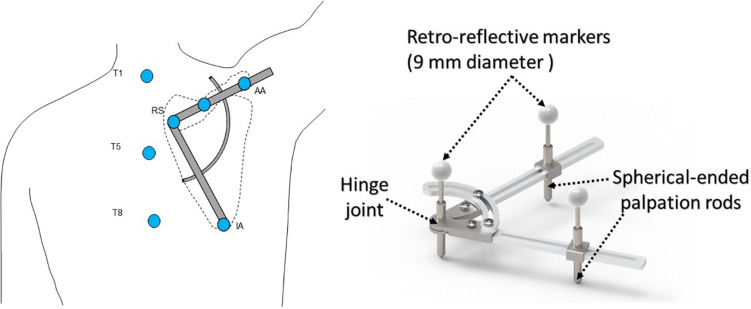
Schematic diagram showing a subject with the scapular locator to measure the scapular kinematics during arm elevation in the scapular plane. The palpation rods were adjusted to fit over the root of the scapular spine (RS), the acromial angle (AA), and the inferior angle (IA) of the scapula. Infrared retro-reflective markers were also attached to the bony landmarks of the trunk, namely the spinal process of the 1st (T1), the 5th (T5), and the 8th thoracic vertebra (T8).

In a gait laboratory, each subject was seated on a height-adjustable chair with the left shoulder against a support fixed to the chair to ensure that the trunk remained vertical and stationary (Kuo et al., 2018). Six infrared retro-reflective markers were attached to the bony landmarks of the trunk to determine its pose in space, namely the spinal processes of the 1st, 5th, and 8th thoracic vertebra (i.e., T1, T5, and T8), xiphoid, sternal notch, and mid-body of the sternum. A compass-like scapular locator with two arms rotating about a hinge joint and three spherical-ended palpation rods was used to track the pose of the scapula (Kuo et al., 2018; Figure 2). The palpation rod for the RS was fixed to and aligned with the hinge joint. The palpation rod for the AA was adjustable along a slot on one arm. The position of the third palpation rod could be adjusted to fit over the IA by moving the rod along the slot on the other arm and/or by changing the angle of the hinge joint (Kuo et al., 2018). The positions of the bony landmarks were each measured by a retro-reflective marker with a 9 mm diameter attached to the top end of each palpation rod. Four markers were attached to the right humerus (i.e., H1–H4). 3D coordinates of the markers were measured using a 7-camera motion capture system (Vicon 512, Oxford Metrics Group, United Kingdom) at 120 Hz.

Each subject then performed static shoulder elevations at 0°, 30°, 60°, 90°, 120°, and 150° in the sagittal, frontal, and scapular plane in a random order. At each elevation position, the testing rater assisted the subject in positioning the upper limb in the desired shoulder elevation position, confirmed using a goniometer. The testing rater then palpated the AA and RS, placed the locator over the two landmarks, and then rotated the locator to make the third rod contact with the IA. Once the locator was placed on the scapula in the correct position, the 3D coordinates of the markers on the locator and those on the trunk and humerus were measured by the motion capture system, which completed a trial of measurement. Each subject was tested four times (trials) for each randomized order of the elevation positions using a counterbalanced measures design.

The trunk, humerus, and scapula were each embedded with an orthogonal coordinate system with the positive *x*-axis directed anteriorly, the positive *y*-axis superiorly, and the positive *z*-axis to the right. Angular positions of the scapula were calculated using a Cardanic rotation sequence (*y*-*x*-*z*), while those for the humerus relative to the trunk were computed using a *y*-*z*-*y* sequence. Values of the rotations and translations of the scapula and clavicle relative to their respective initial positions (i.e., 0° of shoulder elevation) and the amount of humeral elevation relative to the trunk were extracted for statistical analysis.

For statistical analysis, the values of the angles of the GH joint, scapula, and the clavicle relative to the trunk were obtained for both shoulders, each trial, and each subject. All the calculated variables were checked for normality of distribution using a Shapiro–Wilk test before the subsequent *t*-tests. For each variable, data from the six trials were averaged, and the between-group differences were tested for both the affected- and unaffected-side using independent *t*-tests, and the between-side differences were tested using paired *t*-tests. All statistical analyses were performed using SPSS version 20 (SPSS Inc., Chicago, IL, United States). The significance level was set at 0.05 for all tests.

## Results

**FIGURE 3:**
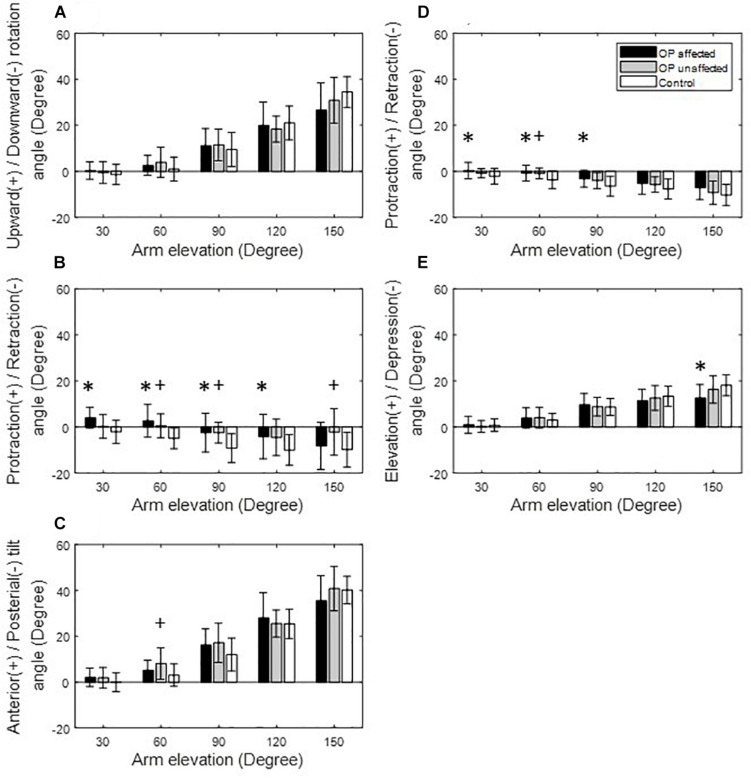
Means and standard deviations of the kinematic components of the scapula **(A–C)** and clavicle **(D,E)** for the affected side (black bars), unaffected side (gray bars) of the patients, and for healthy controls (white bars) during arm elevation in the frontal plane. Symbol “^∗^” indicates a significant difference between the affected side and healthy controls. Symbol “+” indicates a significant difference between the unaffected side and healthy controls.

**FIGURE 4:**
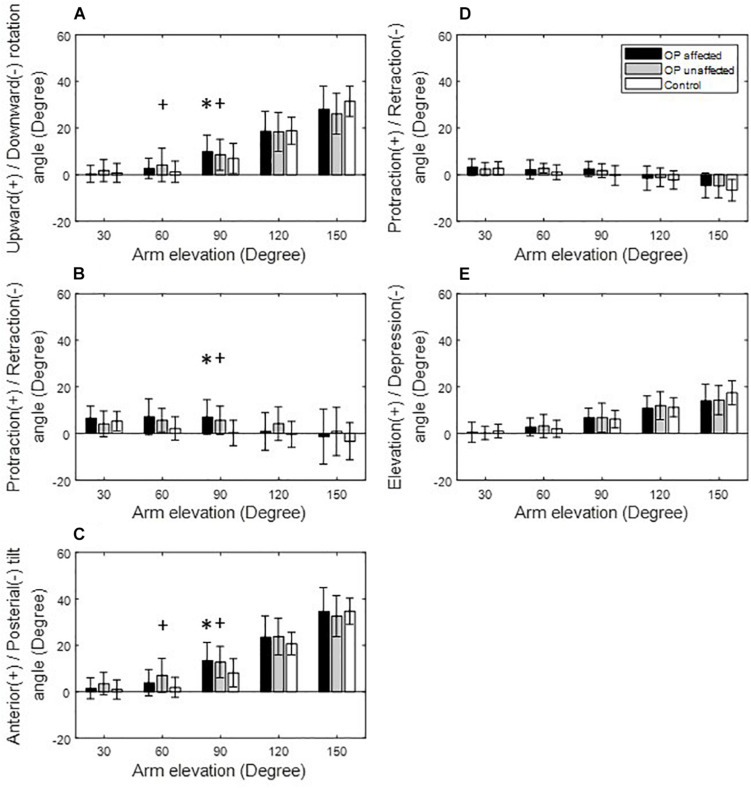
Means and standard deviations of the kinematic components of the scapula **(A–C)** and clavicle **(D,E)** for the affected side (black bars), unaffected side (gray bars) of the patients, and for healthy controls (white bars) during arm elevation in the scapular plane. Symbol “^∗^” indicates a significant difference between the affected side and healthy controls. Symbol “+” indicates a significant difference between the unaffected side and healthy controls.

**FIGURE 5:**
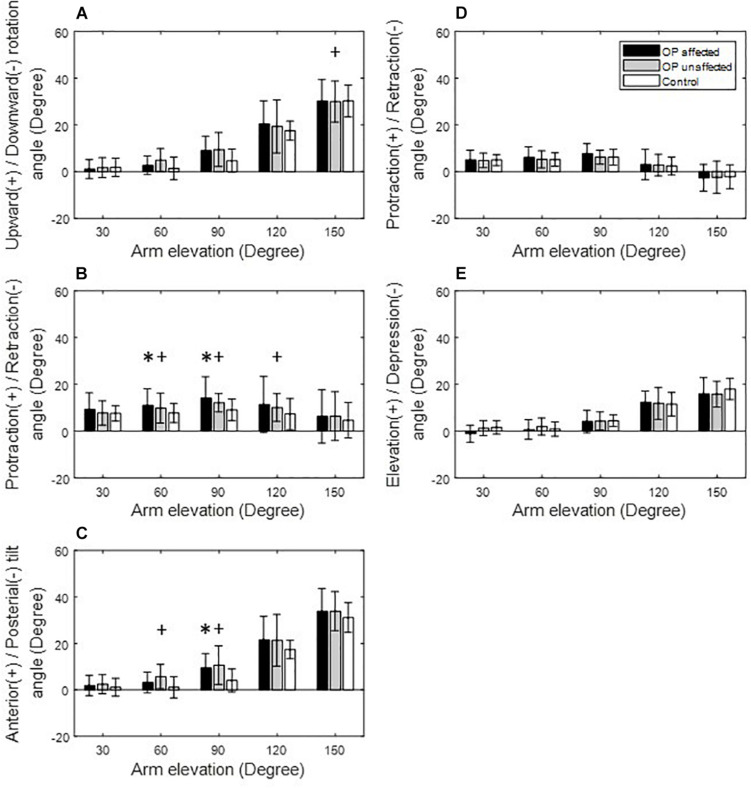
Means and standard deviations of the kinematic components of the scapula **(A–C)** and clavicle **(D,E)** for the affected side (black bars), unaffected side (gray bars) of the patients, and for healthy controls (white bars) during arm elevation in the sagittal plane. Symbol “^∗^” indicates a significant difference between the affected side and healthy controls. Symbol “+” indicates a significant difference between the unaffected side and healthy controls.

The subjective outcome measures for the surgery group were all within normal ranges at the time of the motion experiments [DASH score: 4.48 (5.28); Constant score: 96.57 (5.17); VAS score: 0.5 (0.65); and ASES score: 97.09 (3.00)]. No significant between-side differences in the clavicle length [affected: 161.1 (20.1) mm; unaffected: 160.0 (17.3) mm; and *p* = 0.75] or maximum arm elevation [affected: 155.5 (7.6) mm; unaffected: 153.3 (7.9) mm; and *p* = 0.33] were found in the surgical group. Similar scapulothoracic and claviculothoracic kinematics between the affected and unaffected sides of the surgical patients were found in all kinematic components for all test positions (*p* > 0.05; Figures 3, 4, 5). Similar maximum arm elevations (range of motion) were also found between the Surgical and Control groups [Control: 154.8 (5.8) mm; affected: *p* = 0.81; and unaffected: *p* = 0.55].

**FIGURE 6:**
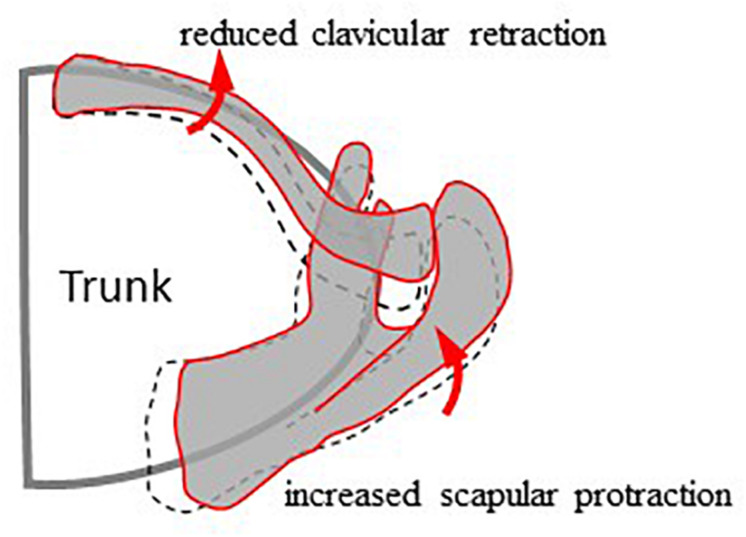
Schematic diagram showing the different kinematics of the scapula and clavicle between the surgical (red lines) and healthy control (black dash lines) groups during arm elevation in the frontal plane. Compared to healthy controls, the surgical group showed reduced clavicular retraction and increased scapular protraction during frontal plane arm elevations while maintaining unaltered scapular rotation and tilt.

During arm elevation in the frontal plane, the surgical group showed significantly reduced clavicle retraction at low elevation angles (i.e., less than 90°) and reduced clavicle elevation at 150° of arm elevation compared to the healthy group (Figures 3, 6). The surgical group also showed significantly increased scapular protraction at 30° and 60° of arm elevation, and significantly less scapular retraction at 90° and 120° arm elevation angles (Figures 3, 6). During arm elevation in the scapular plane, the surgical group showed significantly increased scapular protraction at 60° of arm elevation and significantly increased scapular protraction and anterior tilt at 90° of arm elevation when compared to the healthy group (Figure 4). During arm elevation in the sagittal plane, compared to the healthy group, the surgical group showed significantly increased lateral rotation, protraction, and anterior tilt of the scapula at 90° of arm elevation (Figure 5). No significant between-group differences were found in clavicular kinematics (Figure 5).

## Discussion

The current study aimed to quantify the effects of internal fixation for mid-shaft clavicle fractures on the 3D bone poses of the shoulder complex during multi-plane elevations. The patients were found to have recovered general shoulder function by the time of the motion experiment, as indicated by the normal ranges of the subjective outcome measures. They also showed similar clavicle lengths, maximum arm elevations, and scapulothoracic and claviculothoracic kinematics between the affected and unaffected sides. The improved functional outcome and recovery of shoulder kinematics are most likely a result of the successful restoration of the length of the clavicle after mid-shaft clavicle fracture reduction. Previous studies indicated that the decrease in the clavicle length following a surgical treatment could result in functional deficits of the shoulder (Rasmussen et al., 2011; Robinson et al., 2013). The observed bilateral symmetry in most kinematic components suggests that the surgical treatment was successful in not only reconstructing the form of the fractured clavicle but also restoring the shoulder’s range of motion and most of the shoulder kinematics to be similar to those of the unaffected side. However, residual deviations in the transverse plane components of the scapular and clavicular kinematics remained on the affected side and were also found on the unaffected side. These changes appeared to be compensatory actions within the affected shoulder complex for the overall function. The presence of similar changes on the unaffected side suggests a trend of symmetrical bilateral compensation.

During frontal plane arm elevation, the patients showed increased scapular protraction at lower elevation angles when compared to the healthy controls while keeping the scapular rotation and tilt unaltered. Since the shoulder complex is a kinematic mechanism formed by the clavicle, scapula, and the humerus moving over the thorax with precise coordination (Kontaxis et al., 2009; Cutti et al., 2014; Su et al., 2016; Merolla et al., 2019), any morphological or kinematic changes in any of the skeletal components of the shoulder complex would be expected to lead to compensatory kinematic changes of one or more of the individual components to maintain a functional shoulder complex (Kibler and Sciascia, 2010; Merolla et al., 2019). In the current patient group, the surgically fixed clavicle appeared to have a residual effect that tended to increase its protraction or reduce retraction when performing arm elevation in the frontal plane, further leading to increased scapular protraction for a functional shoulder complex. It is noted that these compensatory changes occurred mainly in the transverse plane, minimizing the effects on the main motion component and the overall range of arm elevation in the frontal plane.

Compensatory changes for arm elevation in the other planes were different, reflecting the 3D nature of the shoulder complex. The ranges of the observed scapular changes during frontal plane arm elevation were reduced to arm elevations at 60° and 90° when performing elevations in the scapular and sagittal planes, respectively, without altering the clavicular kinematics. These results suggest that the observed kinematic changes of the scapula in the patients would be present in activities of daily living involving shoulder elevations ranging between 60 and 90 in the frontal, sagittal, and scapular planes, such as eating with a spoon, answering the phone and brushing same side of the head. Altered scapular kinematics and thus the changed actions of the muscles during such repeated daily activities may have long-term negative consequences on the shoulder functions (Kibler et al., 2013). Considering the kinematic geometry of the shoulder complex, it is clear that arm elevations in the sagittal and scapular planes tended to protract the clavicle and scapula and tilt the scapula anteriorly. Thus, the effects of the retraction limitations of the clavicle were reduced. The current findings suggest that the reduced clavicular retraction led to compensatory changes of the scapular kinematics, which may be the underlying mechanism responsible for the scapular dyskinesis associated with the previously reported compromised functional performance in patients with surgically treated mid-shaft clavicle fractures (Rundquist and Ludewig, 2004; Mirzatolooei, 2011; Shields et al., 2015).

The current results support the suggestions in the literature that displaced mid-shaft clavicle fractures should be surgically fixed to prevent clavicle malunion and for early recovery of shoulder function (Hill et al., 1997; Robinson et al., 2004; Bernstein, 2007). It is suggested that the kinematics of the shoulder complex in different elevation planes should be monitored for any sign of compromised integrated motions of the individual bones following surgical treatment. The current rehabilitative training protocol focused mainly on early range-of-motion mobilization and active range-of-motion and strengthening exercises on the affected side, dosages of which were modified along with the patient’s recovery. Without prior knowledge of the bilateral symmetrical scapular kinematic changes observed in the current study, the therapists might not emphasize relevant muscular training on both sides to reduce the bilateral deviations. The results of the current study suggest that rehabilitative training such as muscle strengthening and synergy stability training may be needed, not only on the affected side but also on the unaffected side, to improve the movement control of the shoulder complex for reducing the bilateral residual kinematic deviations of the scapula during humeral elevation.

The current study was the first in the literature to quantify the effects of internal fixation for mid-shaft clavicle fractures on the 3D bone poses of the shoulder complex during multi-plane elevations. This was partly because of the difficulties in the measurements of the scapular kinematics using skin-mounted markers or sensors, which are prone to soft tissue artifacts associated with the large displacement of the scapula relative to the overlying skin during arm movement (Cutti et al., 2005, 2008; Garofalo et al., 2009; Su et al., 2016; Merolla et al., 2019). In the current study, the benefits of surgical treatment and the possible residual kinematic deviations of the scapula during multi-plane arm elevations were identified non-invasively using a computerized motion analysis system with a newly developed three-point scapular locator (Kuo et al., 2018). This approach enabled accurate, non-invasive, and reliable measurement of the poses of the scapula at a large number of arm positions without fatiguing the subject (Kuo et al., 2018). However, this was limited to the scapular kinematics during quasi-dynamic arm movement. The effects of movement speed and dynamic muscle contractions were not considered. For accurate 3D dynamic motions of the scapular during functional activities, medical imaging approaches may be needed (Lu et al., 2008; Lin et al., 2014, 2018, 2020). Further studies will also be required to investigate the effects of bilateral rehabilitation training on shoulder kinematics and identify the efficacy or side-effects of conservative treatment compared to surgical treatment.

## Conclusion

The current study quantified the effects of internal fixation for mid-shaft clavicle fractures on the 3D bone poses of the shoulder complex during multi-plane elevations. The surgical treatment was found to be effective in recovering most of the shoulder kinematic components. However, the reduced clavicular retraction led to compensatory changes of the scapular kinematics, primarily in the transverse plane. Compared to the healthy controls, the patients showed increased scapular protraction at lower elevation angles and reduced scapular retraction at higher elevation angles during frontal plane arm elevations while maintaining unaltered scapular rotation and tilt. The ranges of the observed scapular changes were reduced to arm elevations at 60° and 90° in the scapular and sagittal planes, respectively, without altering the clavicular kinematics. These observed changes were also found on the unaffected side, suggesting a trend of symmetrical bilateral compensation. The current results suggest that shoulder kinematics in multi-plane arm elevations should be monitored for any sign of compromised integrated motions of the individual bones following surgical treatment. It is also suggested that rehabilitative training such as muscle strengthening and synergy stability training may be needed, not only on the affected side but also on the unaffected side, to improve the bilateral movement control of the shoulder complex during humeral elevation.

## Data Availability Statement

The original contributions presented in the study are included in the article/supplementary material; further inquiries can be directed to the corresponding author/s.

## Ethics Statement

The studies involving human participants were reviewed and approved by Research Ethics Committee of National Taiwan University Hospital. The patients/participants provided their written informed consent to participate in this study.

## Author Contributions

L-WH: conceptualization, data curation, resources, and writing – original draft. H-YL: data curation, formal analysis, and writing – original draft. C-HC: data curation, resources, and writing – original draft. T-YC: data curation, formal analysis, and writing – original draft. T-MW: conceptualization and writing – review and editing. T-WL: conceptualization, supervision, writing – original draft, and writing – review and editing. All authors contributed to the article and approved the submitted version.

## Conflict of Interest

The authors declare that the research was conducted in the absence of any commercial or financial relationships that could be construed as a potential conflict of interest.
